# A hospital-based estimate of major causes of death among under-five children from a health facility in Lagos, Southwest Nigeria: possible indicators of health inequality

**DOI:** 10.1186/1475-9276-11-39

**Published:** 2012-08-08

**Authors:** Bamgboye M Afolabi, Cecilia O Clement, Adejuwonlo Ekundayo, Duro Dolapo

**Affiliations:** 1 Health, Environment and Development Foundation, 34 Montgomery Road, Yaba, Lagos, Nigeria; 2 Department of Morbid Anatomy, College of Medicine, University of Lagos, Idi-araba, Lagos, Nigeria; 3 Silverwood Consult, 9 Oyabiyi Street, Yaba, Lagos, Nigeria; 4 Support to National Malaria Programme, SuNMaP, Abia House, Abuja, Nigeria

**Keywords:** Childhood mortality, Respiratory illnesses, Gastrointestinal diseases, Millennium Development Goal Four, Southwest Nigeria

## Abstract

**Introduction:**

Current evidence on the root-causes of deaths among children younger than 5years is critical to direct international efforts to improve child survival, focus on health promotion and achieve Millennium Development Goal 4. We report a hospital-based estimate for 2005-2007 of the major causes of death in children in this age-group in south-west Nigeria.

**Methods:**

We used retrospective data from the intensive care unit of a second-tier health facility to extract the presenting complaints, clinical diagnosis, treatment courses, prognosis and outcome among children aged 6—59months. SPSS-19 was used for data analysis.

**Results:**

Of the 301 children (58% males, 42% females) admitted into the ICU within the period of study, 173 (26%) presented with complaints related to the gastrointestinal system, 138 (21%) with respiratory symptoms and 196 (29%) with complaints of fever. Overall, 708 investigations were requested for among which were full blood count (215, 30%) and blood slides for malaria parasite (166, 23%). Infection ranked highest (181, 31%) in clinicians’ diagnosis, followed by haematological health problems (109, 19%) and respiratory illnesses (101, 17%). There were negative correlations between outcome of the illness and patient’s weight (r=-0.195, p=0.001) and a strong positive correlation between prognosis and outcome of admission (r=0.196, p=0.001). Of the 59 (20%) children that died, presentation of respiratory tract illnesses were significantly higher in females (75%) than in males (39%) (χ²=7.06; p=0.008) and diagnoses related to gastrointestinal pathology were significantly higher in males (18%) than in females (0%) (χ²=4.07; p=0.05). Majority of the deaths (21%) occurred among children aged 1.0 to 1.9years old and among weight group of 5.1-15.0kg.

**Conclusion:**

The major causes of deaths among under-five years old originate from respiratory, gastrointestinal and infectious diseases – diseases that were recognized as major causes of childhood mortality about half a century earlier. Realization of MDG4 - to reduce child mortality by two-thirds – is only possible if the government and donor agencies look beyond the health sector to find hidden causative factors such as education and housing and within the health sector such as vibrant maternal, new-born, and child health interventions.

## Introduction

Statistics detailing infant and child mortality, especially in sub-Saharan Africa (SSA) are staggering. Though child mortality has been declining worldwide as a result of socioeconomic development and implementation of child survival interventions, yet 8·8 million children die every year before their fifth birthday [1]. Globally, substantial headway has been made towards the achievement of MDG4 as the number of under-fives deaths has declined from more than 12 million in 1990 to 7.6 million in 2010 [2]. In SSA, the average annual rate of reduction in under-five mortality has accelerated, doubling from 1990-2000 to 2000-2010. UNICEF also reports that approximately 50% of under-five deaths occur in only five countries including India, Nigeria, Democratic Republic of Congo, Pakistan and China, that India (22%) and Nigeria (11%) together account for a third of all under-five deaths, that 70% of under-five deaths occur within the first year of life and that the highest rates of child mortality are still in SSA [2].

Disadvantaged children – economically disadvantaged and medically underserved or those that are difficult-to-contact and living in hard-to-reach locations – may face many obstacles to accessing and receiving effective health services such as health promotion, disease prevention, early detection and high-quality medical treatment [3]. Children who live in metropolitan settings in SSA, who are not difficult to contact, not medically underserved and not living in hard-to-reach geographical settings often experience suboptimal health outcomes [4]. These children face such barriers as inadequate purchasing power of parents or guardians, being uninsured or underinsured or living within a weak health system [5,6]. Furthermore, social determinants of health such as rapid urbanization together with the poor economic circumstances [7] are almost completely overlooked as key obstacles to improving integrated action by health care sector on child health in Nigeria. Although African leaders, including Nigeria, adopted the MDGs as a tool within their wider development planning framework, and though significant progress has been made by some sub-regions and countries that adopted MDGs, Africa fared worst among the world’s regions, showing the slowest progress overall and suffered reverses in some crucial areas such as living conditions and life expectancy [8]. Despite the fact that Nigeria, an oil-rich country, which has achieved high growth rates in the last five years – approximately 6.5% annually and which spends a large proportion of its gross domestic product (GDP) on government expenditure (33.8% of GDP in 2010) – still 54% of her population still lives in poverty [9]. Improving social protection would be one way to address this policy paradox. A recent World Health Organization report warns that the health related millennium development goals (MDGs) will not be met without a dramatic increase in investments in national health systems [10]. Over the past 20years, statistics about child mortality in Nigeria indicates that 115 of children die before their fifth birthday [11], with under-five mortality rate of 140 deaths per 1000 live births for 1995-1999 period [12] and overall under-five mortality rate of 201 deaths per 1000 live births [13]. Data from multi-cause proportionate mortality models indicate that infectious diseases caused 68% of the deaths in children younger than 5years old [14]. If, as suggested by Rahman and Sarkar [15], infant and child mortality rates reflect a country’s socio-economic development and quality of life, then the high infant and child mortality rates reflects a low socio-economic development and a poor quality of life in a wealthy, oil-producing and nation like Nigeria. This situation must change. As a first step toward change, some questions need to be answered: How is Nigeria performing with respect to health and health equity? What are the key obstacles to improving integrated action by health care sectors on the social determinant of health? The main concern of this study is to examine leading causes of death among males and female under-fives. This study also attempts to relate child mortality in Nigeria to attention not being paid by the government to social determinants of health.

## Methodology

### Study area characteristics

Nigeria, located in the West Africa, between Benin Republic to the west and Cameroon Republic to the east has a population of about 156 million. It has six geopolitical zones and 36 States including the Federal Capital Territory. It has a total area of 923,770 sq. km of which land covers 910,770 sq. km, a coastline stretching 853km with two principal rivers – Niger and Benue – merging at about mid country and emptying into the Gulf of Guinea, further south. The climate varies between equatorial in the south, tropical in the centre and arid in the north. Southern lowlands merge into central hills and plateau with mountains in the south-eastern part and plains further north. The vital statistics of Nigeria are still a concern to the government and the international community with an Infant Mortality Rate (IMR) of 75 per 1000 live births, Under-five Mortality Rate (U5MR) of 140 per 1000, Maternal Mortality Rate (MMR) of 704 per 100,000 and Crude Death Rate of 14 per 1000. This worrisome statistics accrue from prevalence rate of malaria being 1858 per 100,000, diarrhoea at 896 per 100,000, pneumonia at 208 per 100,000 and measles at 141 per 100,000. Life expectancy at birth is 52.2 years [16].

### Study site

Lagos State, with a daytime population of about 14 million (National Population Council, Census 2000), forms one of the 36 States and Federal Capital Territory that constitute Nigeria. The state has a free malaria treatment programme initiated over 10 years ago which has not been evaluated since inception. The health facility where this survey took place is located on Lagos Island and has been described elsewhere [17]. The health facility has about 80 beds, serving approximately 100,000 residents. Most of its clients come from urban Lagos but occasional referrals are from neighbouring cities, towns and villages. There are at least 30 nurses and 15 clinicians running a 24 hour shift. The facility has administrative, in-patient, out-patient, intensive care and logistics departments. The health facility receives patients from Lagos metropolitan, especially among the immediate surround with it poor housing, unacceptable sanitation and sewage disposal, and poor infrastructures. Patients’ data for this study were retrieved from the intensive care unit (ICU). Patients usually have multiple presentations because of co-morbidity or a single illness having various manifestations.

### Study design

This is a health facility-based retrospective case review of records kept at ICU. Individual ICU admission case file for the reference period (Mar 2005-Feb 2007) were identified and retrieved. Information retrieved into this form included patients hospital number, date of admission, patients biodata, presenting complaints, clinicians’ diagnosis, investigations requested for, prescribed medications and/or procedures, prognosis of the illnesses, outcome and duration of admission.

#### Research problem

Statistics of childhood mortality is worrisome to government and parents are perplexed about the reasons and causes of children dying before they are five years old. Lack of consideration for and responsiveness to social determinants of health may be responsible for high childhood mortality rate in Nigeria. Nigerian government should emulate the OECD countries and some oil-rich Arab nations in addressing health by reducing health inequity and inequality from the perspective of social determinants of health. This study attempts to produce a league table of major diseases which kill under-fives in south-west Nigeria but which can be easily prevented not by expensive biomedical imagination but by easily affordable health promotion. This will catapult the nation to a rapid progress towards the achievement of MDG 4 by 2015. Otherwise, Nigeria, unlike some other sub-Saharan countries, may not readily achieve this global target on time.

#### Research question

Since most health sector plans have not yielded results fast enough, then, what definitive strategy has to be in place to reduce childhood mortality in Nigeria fast enough to attain MDG4 by 2015?

#### Boundary of the study

This study is limited to Nigerian children under the age of five who were admitted into the intensive care unit of a second-tier paediatric hospital in Lagos.

#### Ethics committee approval

Necessary Ethics Committee approval of the Lagos State Health Management Board, Campbell Street, Lagos, Nigeria was secured before carrying out this study. Informed consent was obtained from care-givers where feasible or from representatives of subjects of the study.

### Data collection

Two data collectors were recruited to retrieve data from the hospital medical records. A pro-forma data record form was designed for transcription of data from the medical records. Data were collected over a 3-month period and simultaneously transcribed verbatim into the data record form after close inspection.

### Data analysis

Data of each child was coded for anonymity, ease of reference, avoidance of bias and fed into a lap top computer, cleaned and cross-checked for errors. Analysis of the cleaned data was done using Statistical Package for Social Sciences version 19 for Windows software. For easier analysis, age (years) was grouped (age group) into four categories viz. (i)1-1.9, (ii) 2-2.9, (iii) 3-3.9 and (iv) 4-4.9; weight (kg) was categorized into (i) ≤5 (ii) 5.1-10 (iii) 10.1-15 and (iv) 15.1-20. Diagnosis of illness entities was coded from 1-11 where 1=respiratory, 2=hematology, 3=gastrointestinal, 4=nutritional, 5=malaria, 6=measles, 7=cardiac, 8=nervous, 9=meningitis, 10=septicaemia and 11=others. Prognosis was coded into 1=good, 2=fair, 3=poor, 4=very poor and 5=very, very poor. Outcome of admission into ICU was graded in a scale of 6 where 1=full recovery and discharged home, 2=absconded, 3=referred to another health facility, 4=discharged against medical advice (DAMA), 5=still on admission and 6=patient died. Morbidity and mortality data were analysed descriptively obtaining frequencies and percentages, and inferentially using chi-square test to determine associations, where appropriate. A p-value ≤0.05 was regarded as significant. Pearson’s coefficient of correlation (r) was determined for all normally distributed variables while Spearman’s correlation coefficient was determined for variables without normal distribution. Gamma value was used to determine strength and association of ordinal data on a Likert scale. The significance of association/correlation was determined by student’s *T*-test (2-tailed) and significance level was set at ≤0.05. Differences were considered significant when p was ≤0.05. Tables, charts and graphs were used to present data.

## Results

Medical records of 301 children (173 males and 128 females) who were admitted into the intensive care unit (ICU) of a second-tier paediatric health facility between March 2005 and February 2007 were reviewed.

### General characteristics of study children

Table 1 reports the general characteristics of the study children by age-group and by gender differentiation. There was no significant difference in the number of children in the various age-groups, though majority (148, 49%) were 1-1.9 years and the least number (25, 8%) were aged 4-4.9 years, an indication that younger children are more susceptible to various factors that bring about morbidity and mortality among this group of people. Males predominated admission into ICU than females. As expected, weight varied significantly according to age group (F = 115.04, p = 0.000).

**Table 1:** Characteristics of under-fives admitted into intensive care unit of a secondary health facility in the survey (2004-2007)

	**ALL**	**By gender**		**By age (years)**			
		**Male**	**Female**	**1-1.9**	**2-2.9**	**3-3.9**	**4-4.9**
Number	301	173	128	148	73	55	25
%		57.5	42.5	49.2	24.3	18.3	8.3
-Male	-	-	-	89	42	31	11
-%	-	-	-	51.4	24.3	17.9	6.4
-Female	-	-	-	59	31	24	14
-%	-	-	-	43.1	24.2	18.8	10.9
				*χ*^2^=2.31, df=3, p=0.51			
Age (year)							
-Mean	2.1	2.0	2.1	1.2	2.2	3.2	4.1
-±SEM	0.06	1.0	1.0	0.02	0.03	0.03	0.03
-Min.	1.0	1.0	1.0	1.0	2.0	3.0	4.0
-Max.	4.5	4.5	4.0	1.9	2.8	3.5	4.5
				F-statistics=1512.90, p=0.000			
Weight (kg)							
-Mean	10.0	10.2	9.8	7.9	10.3	12.5	14.8
-±SEM	3.7	4.2	2.9	0.16	0.26	0.29	0.61
-Min.	2.5	2.5	3.8	3.1	2.5	5.2	7.5
-Max.	39.8	39.8	18.7	15.0	15.0	17.0	20.0
				F-statistics=115.04, p=0.000			
Temperature °C							
-Mean	37.2	37.2	37.2	37.2	37.3	37.1	37.2
-±SEM	0.05	0.8	0.9	0.07	0.10	0.10	0.13
-Min.	35.0	35.0	35.2	35.0	36.0	36.0	36.0
-Max.	40.2	40.2	39.6	39.8	40.2	39.6	39.0
				F-statistics=0.319, p=0.811			

### Presenting complaints in the ICU

**Figure 1:**
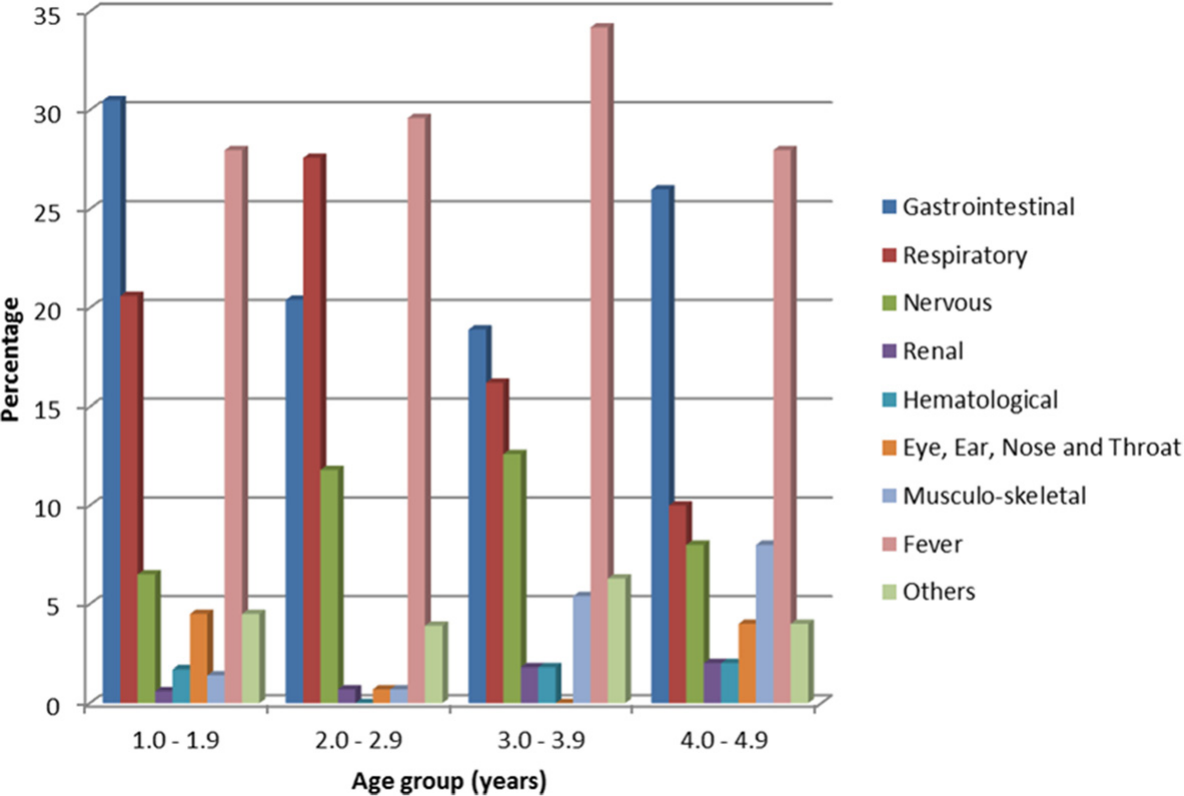
Per cent systemic and general complaints at intensive care unit relative to age group (years).

**Figure 2:**
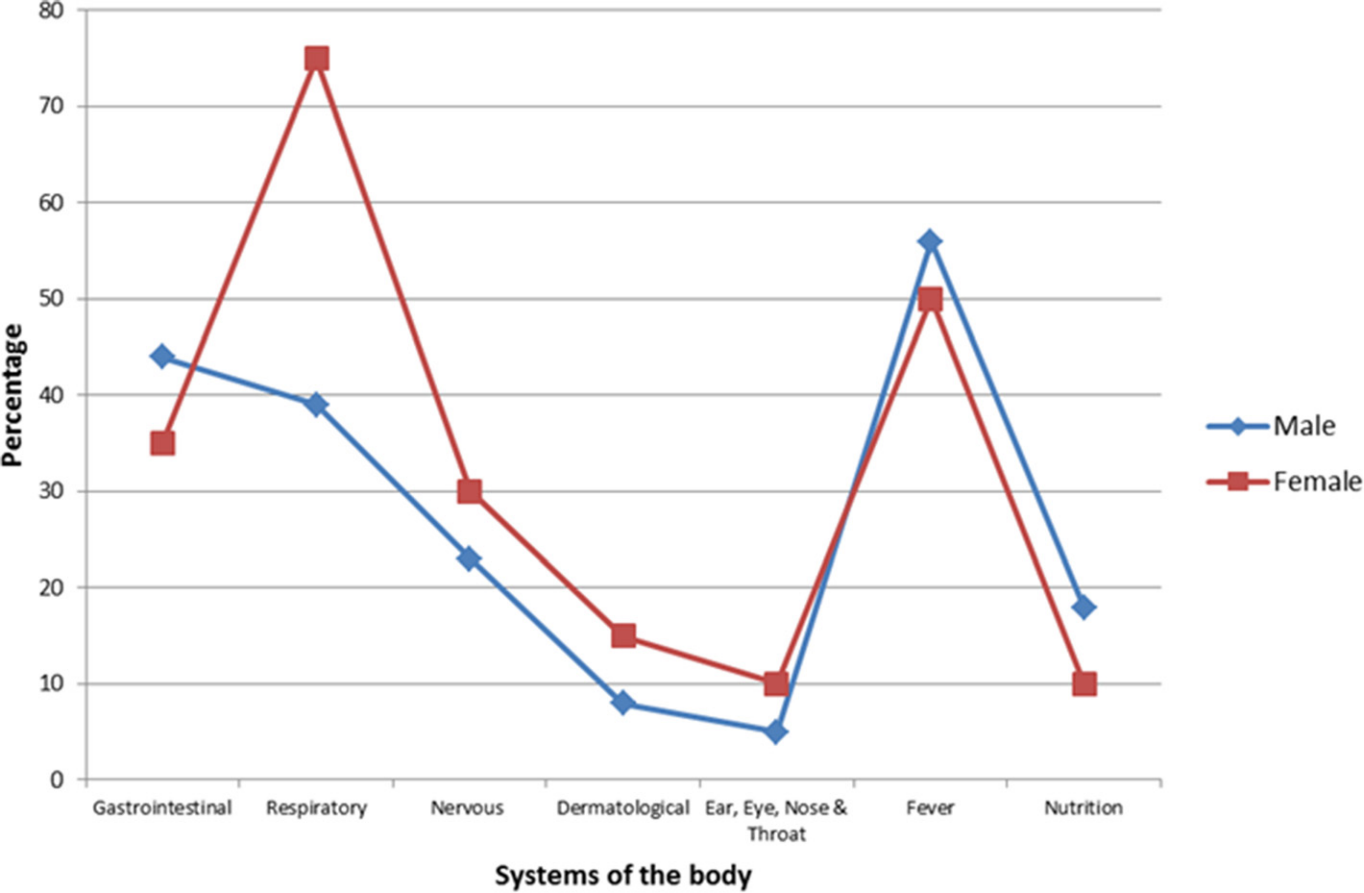
Presenting complaints of survey children that died in intensive care unit of a secondary healthcare facility in Lagos.

Table 2 details the major presenting complaints at ICU according to patients’ age groups. In all, there were 667 complaints made at presentation at the ICU with fever having the highest frequency (196, 29%) followed by complaints relative to the gastrointestinal system (173, 26%), the respiratory system (138, 21%) and the nervous system (59, 9%). Significant differences were observed in the proportions of children aged 1-1.9 and 2-2.9 that presented with gastrointestinal symptoms (OR = 1.71 [1.06-2.77]; χ² = 5.5; p = 0.02) or nervous symptoms (OR = 0.52 [0.26-1.04]; χ² = 4.08; p = 0.04) and when 1-1.9 year age group was compared to 3-3.9 year age group that presented with gastrointestinal (OR = 1.88 [1.08-3.30]; χ² = 5.7; p = 0.02) or nervous symptom (OR = 0.48 [0.23-1.03]; χ² = 4.3; p = 0.04). Of the 708 investigations ordered for by the clinicians, full blood count had the highest frequency (215, 30%) followed by investigations for malaria parasitaemia (166, 23%) and erythrocyte sedimentation rate (ESR) (64, 9%). Figure 1 is a graphical representation of the pre-mortality presentation of illnesses affecting various systems of the body in different age groups indicating that in all age groups, fever as well as gastrointestinal and respiratory diseases are the dominant illnesses that brought these children to the ICU. Figure 2 separates these children into males and females and shows that a significant proportion of female children (15, 75%; χ² = 7.06, p = 0.008) presented more with symptoms of respiratory diseases than males (15, 39%).

**Table 2:** Major presenting complains of under-fives admission in intensive care unit and clinical investigations requested for (2005-2006 study)

	**Age (years)**				
	**1.0 – 1.9**	**2.0 – 2.9**	**3.0 – 3.9**	**4.0 – 4.9**	
	(n=148)	(n=73)	(n=55)	(n=25)	
	**No. (%)**	**No. (%)**	**No. (%)**	**No. (%)**	**Total (%)**
**Common complaints (n=667)**	**354 (53.1)**	**152 (22.8)**	**111 (16.6)**	**50 (7.5)**	**667 (100)**
- Gastrointestinal system	108 (62)*!	31*	21!	13	173 (25.9)
- Respiratory system	73 (53)	42	18	5	138 (20.7)
- Dermatological system	16 (53)	7	3	4	30 (4.5)
- Eye, Ear, Nose and Throat	6	1	0	2	9 (1.3)
- Nervous system	23 (39)**!!	18**	14!!	4	59 (8.8)
- Renal system	2	1	2	1	6 (0.9)
- Hematological system	6	0	2	1	9 (1.3)
- Musculo-skeletal system	5	1	6	4	6 (2.4)
- Fever	99 (51)	45	38	14	196 (29.4)
- Others	16	6	7	2	31 (4.6)
**Common investigations (n=708)**	**359 (50.7)**	**166 (22.4)**	**130 (18.4)**	**53 (7.5)**	**708 (100)**
- Full blood count	111	50	37	17	215 (30.4)
- Malaria parasite	89	36	32	9	166 (23.4)
- Chest x-ray	36	11	6	0	53 (7.5)
- ESR	35	9	11	9	64 (9.0)
- Electrolyte/urea/creatinine	23	15	9	3	50 (7.1)
-Cerebrospinal fluid	15	8	3	3	29 (4.1)
-PCV	9	20	19	8	56 (7.9)
- WBC/Differentials	8	1	2	0	11 (1.6)
- Grouping/cross-matching	6	5	3	1	15 (2.1)
- Urinalysis/MCS	3	3	1	0	7 (1.0)
- Others	24	8	7	3	42 (5.9)

*Chi-square test of significance in proportion that presented with gastrointestinal symptom in agegroups1 and 2: OR=1.71 (1.06-2.77); χ²=5.5; p=0.02.

**Chi-square test of significance in proportion that presented with nervous symptom in agegroups1 and 2: OR=0.52 (0.26-1.04); χ²=4.08; p=0.04.

!Chi-square test of significance in proportion that presented with gastrointestinal symptom in agegroups1 and 3: OR=1.88 (1.08-3.30); χ²=5.7; p=0.02.

!!Chi-square test of significance in proportion that presented with nervous symptom in agegroups1 and 3: OR=0.48 (0.23-1.03); χ²=4.3; p=0.04.

### Clinicians’ diagnoses

**Figure 3:**
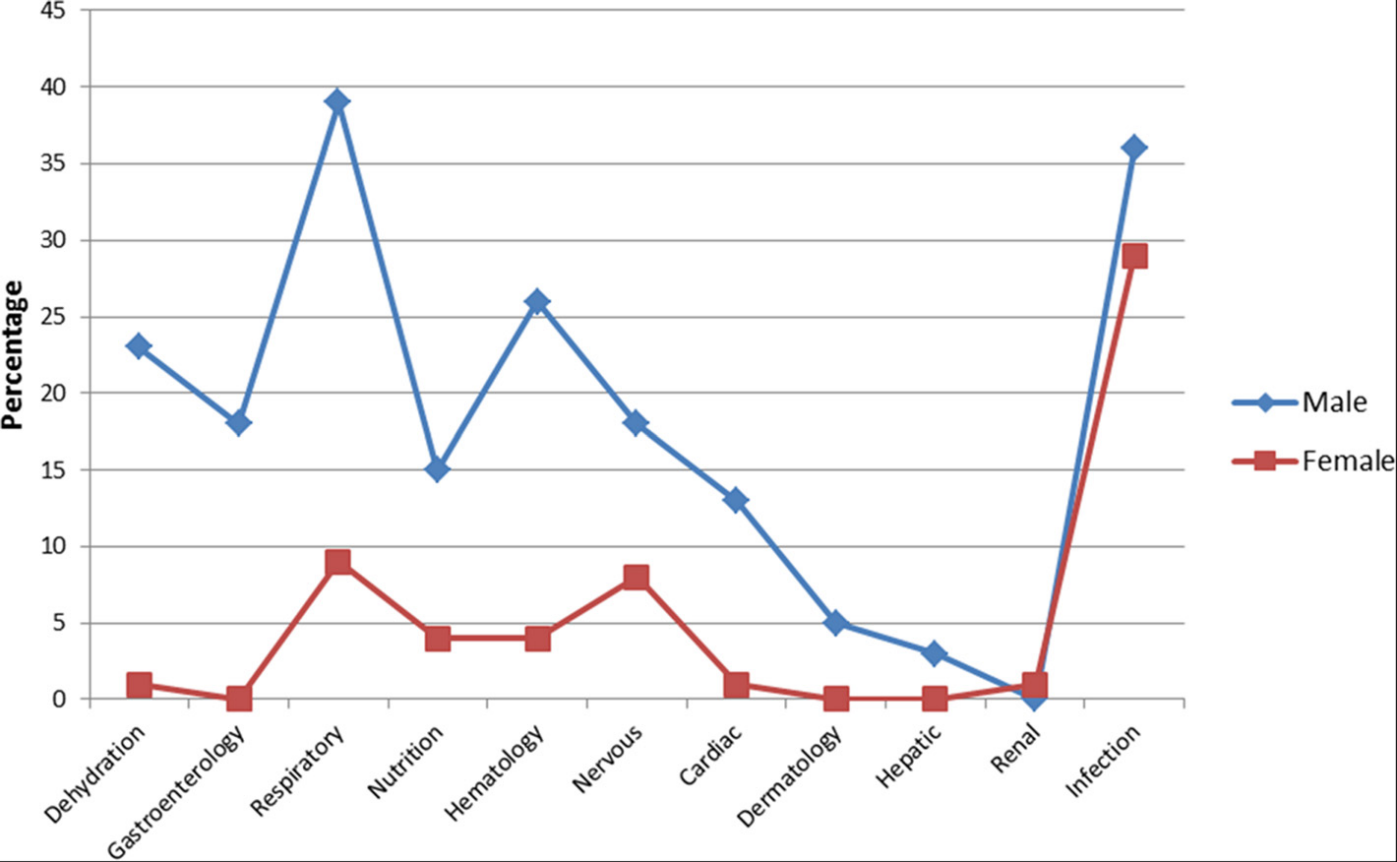
Clinicians’ diagnoses of survey children prior to death at the intensive care unit of a secondary healthcare facility in Lagos.

Of the 587 diagnoses made by the attending clinicians (Table 3), diagnosis of infection was significantly higher among children aged 1-1.9 years (OR = 0.60 [038-0.96], χ² = 5.2, p = 0.02) compared to those aged 2-2.9 years and more so when compared with those aged 3-3.9 years (OR = 0.39 [0.23-0.66], χ² = 14.80, p = 0.0001). This comparison was also significant between 1-1.9 and 4-4.9 years old (OR = 0.45 [0.22-0.92], χ² = 5.8, p = 0.02). Among the infectious diseases diagnosed, malaria was the highest (131, 72%) followed by measles (45, 25%). The diagnosis of gastrointestinal illness was significantly higher among age group 1-1.9 compared with those aged 2.2.9 (OR 5.72 [1.65-23.73], χ² = 10.2, p = 0.001) and those aged 3 = 3.9 (χ² = 11.5, p = 0.0007); the diagnosis of respiratory tract illness was also significantly higher among those aged 1-1.9 years compared with those aged 4-4.9 years (OR 4.28 [0.97-26.37], χ² = 4.53, p = 0.03), the diagnosis of nutritional deficiencies was significantly higher in age group 1-1.9 compared to 3-3.9 (χ² = 5.78, p = 0.02) and the diagnosis of haematological problems was equally significantly higher among age group 1-1.9 than age group 2-2.9 (OR 0.51 [0.30-0.87], χ² = 7.08, p = 0.008) and age group 3-3.9 (OR 0.41 [0.23-0.74], χ² = 10.4, p = 0.001). A substantial number of males were diagnosed with gastrointestinal tract illness (7, 18%; Fisher’s exact p = 0.05) than females (0, 0%) (Figure 3).

**Table 3:** Clinicians’ diagnoses by age groups of under-fives in intensive care unit in metropolitan Lagos Nigeria (2005-2006 study)

	**Age (years)**				**Total (%)**
	**1.0 – 1.9**	**2.0 – 2.9**	**3.0 – 3.9**	**4.0 – 4.9**	
	**(n = 148)**	**(n = 73)**	**(n = 55)**	**(n = 25)**	
	**No. (%)**	**No. (%)**	**No. (%)**	**No. (%)**	
**Diagnosis (n=587)**	**317**	**137**	**92**	**41**	**587 (100)**
Nutritional	19	3	0	0	23 (3.9)
Respiratory	57	26	16	2	101(17.2)
Cardiac	17	4	3	1	25 (4.3)
Dermatological	3	0	0	0	3 (0.5)
Nervous	16	13	9	5	43 (7.3)
Hematological	44	33	26	6	109 (18.6)
Hepatic	0	1	0	1	2 (0.3)
Renal	2	1	2	1	6 (1.0)
Gastrointestinal	36	3	0	3	42 (7.2)
Musculo-skeletal	4	0	0	2	6 (1.0)
Endocrine	2	0	0	1	3 (0.5)
Inflammatory	2	1	0	0	3 (0.5)
Dehydration	27	2	0	1	30 (5.1)
Fever of undetermined origin	1	0	0	0	1 (0.2)
Infection	76	47	41	17	181 (30.8)
- Malaria	46	35	36	14	131 (72.4)
- Measles	29	11	3	2	45 (24.9)
- Typhoid	0	0	1	0	1 (0.6)
- Cholera	1	0	1	0	1 (0.6)
- Tetanus	0	1	0	1	2 (1.1)
Others	11	3	4	1	19 (3.2)

Table 4 addresses mainly the clinicians’ diagnoses based on initial presentation at ICU before death occurred among specific male and female patients, bearing in mind that more often these children presented with multiple complaints. Fever, a cross-cutting symptom, was more prevalent among males (22, 56%) than females (10, 50%) though the difference was not significant. The proportion of female children that presented with respiratory illnesses (15, 75%) was significantly higher (OR = 0.21 [0.05-0.79], χ² = 7.1, p = 0.008) than males (15, 38.5%). Among the children that died, the diagnosis of gastrointestinal illness was significantly higher among males than among females (χ² = 4.1, p = 0.04).

**Table 4:** Mortality by age-group, sex, presentation and preliminary diagnosis of under-fives in intensive care unit (ICU) in metropolitan Lagos Nigeria (2005-2006 study)

	**Age group (years)**				
	**1.0 – 1.9**	**2.0 – 2.9**	**3.0 – 3.9**	**4.0 – 4.9**	**Total**
	**(n=148)**	**(n=73)**	**(n=55)**	**(n=25)**	**(n=301)**
**Died**	**No. (%)**	**No. (%)**	**No. (%)**	**No. (%)**	**No. (%)**
	**31 (20.9)**	**14 (19.2)**	**10 (18.2)**	**4 (16.0)**	**59 (18.3)**
- male	22 (70.9)	9 (64.3)	7 (70.0)	1 (25.0)	39 (66.1)
- female	9 (19.1)	5 (35.7)	3 (30.0)	3 (75.0)	20 (33.9)
**Presentation at ICU**					
-Male					
○ Gastrointestinal	16 (73)	0 (0)	1 (14)	0 (0)	17 (43.6)
○ Respiratory*	7 (32)	7 (78)	1 (14)	0 (0)	15 (38.5)
○ Nervous system	3 (14)	3 (33)	3 (43)	0 (0)	9 (23.1)
○ Dermatological system	2 (9)	1 (11)	0 (0)	0 (0)	3 (7.7)
○ Eye, Ear, Nose and Throat	2 (9)	0 (0)	0 (0)	0 (0)	2 (5.1)
○ Fever	8 (36)	7 (78)	6 (86)	1 (100)	22 (56.4)
○ Nutritional	3 (14)	0 (0)	3 (43)	1 (100)	7 (17.9)
- Female					
○ Gastrointestinal	4 (44)	2 (40)	0 (0)	1 (33)	7 (35.0)
○ Respiratory*	7 (78)	3 (60)	2 (67)	3 (100)	15 (75.0)
○ Nervous system	4 (44)	1 (20)	1 (33)	0 (0)	6 (30.0)
○ Dermatological system	0 (0)	1 (20)	0 (0)	2 (67)	3 (15.0)
○ Eye, Ear, Nose and Throat	0 (0)	0 (0)	1 (33)	1 (33)	2 (10.0)
○ Fever	4 (44)	3 (60)	1 (33)	2 (67)	10 (50.0)
○ Nutritional	0 (0)	1 (20)	0 (0)	1 (33)	2 (10.0)
*Chi-square test of significance in proportion that presented with respiratory symptom in males and in females: OR = 0.21 (0.05-0.79); χ²=7.1; p=0.008:					
**Diagnosis at ICU**					
- Male					
○ Dehydration	8 (36)	1 (11)	0 (0)	0 (0)	9 (23.1)
○ Gastrointestinal	7 (32)	0 (0)	0 (0)	0 (0)	7 (17.9)
○ Respiratory	7 (32)	4 (44)	4 (57)	0 (0)	15 (38.5)
○ Nutritional	5 (23)	1 (11)	0 (0)	0 (0)	6 (15.4)
○ Hematological	5 (23)	1 (11)	2 (29)	1 (100)	10 (25.6)
○ Nervous system	4 (18)	2 (22)	1 (14)	0 (0)	7 (17.9)
○ Cardiac system	2 (9)	2 (22)	1 (14)	0 (0)	5 (12.8)
○ Dermatological	2 (9)	0 (0)	0 (0)	0 (0)	2 (5.1)
○ Hepatic system	0 (0)	0 (0)	0 (0)	1 (100)	1 (2.6)
○ Renal system	0 (0)	0 (0)	0 (0)	0 (0)	0 (0.0)
○ Infection					
Malaria	6 (27)	2 (22)	5 (71)	1 (100)	14 (35.9)
Measles	5 (23)	1 (11)	1 (14)	0 (0)	7 (17.9)
- Female					
○ Dehydration	1 (11)	0 (0)	0 (0)	0 (0)	1 (5.0)
○ Gastrointestinal	0 (0)	0 (0)	0 (0)	0 (0)	0 (0.0)
○ Respiratory	5 (56)	1 (20)	2 (67)	1 (33)	9 (45.0)
○ Nutritional	2 (22)	2 (40)	0 (0)	0 (0)	4 (20.0)
○ Hematological	2 (22)	2 (40)	0 (0)	0 (0)	4 (20.0)
○ Nervous system	3 (33)	2 (40)	1 (33)	2 (67)	8 (40.0)
○ Cardiac	0 (0)	0 (0)	1 (33)	0 (0)	1 (5.0)
○ Dermatological	0 (0)	0 (0)	0 (0)	0 (0)	0 (0.0)
○ Hepatic system	0 (0)	0 (0)	0 (0)	0 (0)	0 (0.0)
○ Renal system	0 (0)	1 (20)	0 (0)	0 (0)	1 (5.0)
○ Infection					
Malaria	4 (44)	3 (60)	1 (33)	2 (67)	10 (50.0)
Measles	3 (33)	2 (40)	0 (0)	2 (67)	7 (35.0)

*Chi-square test of significance in proportion that were diagnosed with gastrointestinal illnesses among males and females: χ² = 4.1; p = 0.04.

### Mean weight of children that died in ICU by age group, gender and weight group (Table 5)

**Table 5:** Comparison of means of weight of children discharged home (Dchd.) and those that died in ICU

	**Age group (years)**									
	**1-1.9**		**2-2.9**		**3-3.9**		**4-4.9**		**Total**	
	Dchd.	Died	Dchd.	Died	Dchd.	Died	Dchd.	Died	Dchd.	Died
Number	90	31	52	14	40	10	19	4	20	59
Mean wt. (kg)	8.2	7.0	10.3	9.6	13.0	11.2	15.1	13.6	10.4	8.7
±SEM	0.2	0.3	0.3	0.8	0.3	0.9	0.5	3.1	0.2	0.4
Min.	4.0	3.8	5.9	2.5	9.6	5.2	11.6	7.5	4.0	2.5
Max.	15.0	11.0	14.0	14.0	17.0	19.0	20.0	19.0	20.0	19.0
df	56.1		15.7		11.3		3.2		87.4	
t	3.4		0.8		1.9		0.5		3.3	
p	0.001		0.43		0.09		0.65		0.002	
***Male children in ICU***										
Number	51	22	30	9	23	7	9	1	113	39
Mean	8.4	7.0	10.9	9.2	13.1	10.5	16.0	10.5	10.6	8.4
±SEM	0.3	0.4	0.4	1.2	0.4	1.1	0.9	-	0.3	0.5
Min.	4.1	4.3	5.9	2.5	10.0	4.1	11.6	5.2	4.1	2.5
Max.	15.0	11.0	14.0	14.0	17.0	20.0	20.0	14.0	20.0	19.0
df	46.36		9.41		7.64		-		63.0	
t	3.3		1.4		2.1		-		3.7	
p	0.002		0.20		0.07		-		0.001	
***Female children in ICU***										
Number	39	9	22	5	17	3	10	3	88	20
Mean	7.9	7.0	9.5	10.4	12.5	12.9	14.2	11.1	9.9	9.3
±SEM	0.3	0.5	0.3	0.8	0.4	0.6	0.5	2.9	0.3	0.7
Min.	4.0	3.8	6.5	8.5	9.5	12.0	12.0	7.5	4.0	3.8
Max.	12.0	8.5	11.0	12.4	16.0	14.0	17.5	16.7	17.5	16.7
df	12.5		4.9		4.3		2.1		26.3	
t	1.7		-1.1		-0.4		1.1		0.8	
p	0.12		0.33		0.67		0.38		0.44	

Table 5 shows the means of weight of children that recovered and discharged home and those that died in the ICU. The mean weight of all the children that died was significantly lower (t- test = 3.26, df = 87.4, p = 0.002) that that of the children discharged. Specifically, it seems that children aged 1-1.9 years contributed more to this statistics (t-test = 3.43, df = 56.07, p = 0.001) than other age groups. There was no significant difference in the mean weight of female children that died and that were discharged in any of the age groups.

### Prognostic values of children in ICU (Table 6)

**Table 6:** Prognostic values of children that died and outcome of admission into the ICU

	**Discharged**				**Died**			
	**Age group (years)**				**Age group (years)**			
	**1-1.9**	**2-2.9**	**3-3.9**	**4-4.9**	**1-1.9**	**2-2.9**	**3-3.9**	**4-4.9**
***Prognosis***				***All studied children***				
-Good	0 (0)	0 (0)	1 (3)	0 (0)	0 (0)	0 (0)	0 (0)	0 (0)
-Fair	3 (3)	3 (6)	0 (0)	2 (11)	0 (0)	1 (7)	0 (0)	0 (0)
-Poor	41 (46)	24 (46)	17 (42)	11 (58)	5 (16)	1 (7)	2 (20)	3 (75)
-Very poor	45 (50)	25 (48)	22 (55)	6 (32)	25 (81)	12 (86)	8 (80)	1 (25)
-Extremely poor	1 (1)	0 (0)	0 (0)	0 (0)	1 (3)	0 (0)	0 (0)	0 (0)
***Male children admitted into ICU***								
-Fair	0 (0)	1 (7)	0 (0)	0 (0)	0 (0)	0 (0)	1 (7)	0 (0)
-Poor	5 (16)	1 (7)	2 (20)	3 (75)	2 (50)	5 (13)	2 (13)	1 (50)
-Very poor	25 (80)	12 (86)	8 (80)	1 (25)	2 (50)	32 (84)	12 (80)	1 (50)
-Extremely poor	1 (4)	0 (0)	0 (0)	0 (0)	0 (0)	1 (3)	0 (0)	0 (0)
***Female children admitted into ICU***								
-Fair	0 (0)	1 (11)	0 (0)	0 (0)	0 (0)	0 (0)	1 (11)	0 (0)
-Poor	4 (18)	1 (11)	2 (29)	0 (0)	1 (33)	4 (15)	2 (22)	0 (0)
-Very poor	17 (77)	7 (78)	5 (71)	1 (100)	2 (64)	21 (81)	6 (67)	1 (100)
-Extremely poor	1 (5)	0 (0)	0 (0)	0 (0)	0 (0)	1 (4)	0 (0)	0 (0)

( ) = %.

**Figure 4:**
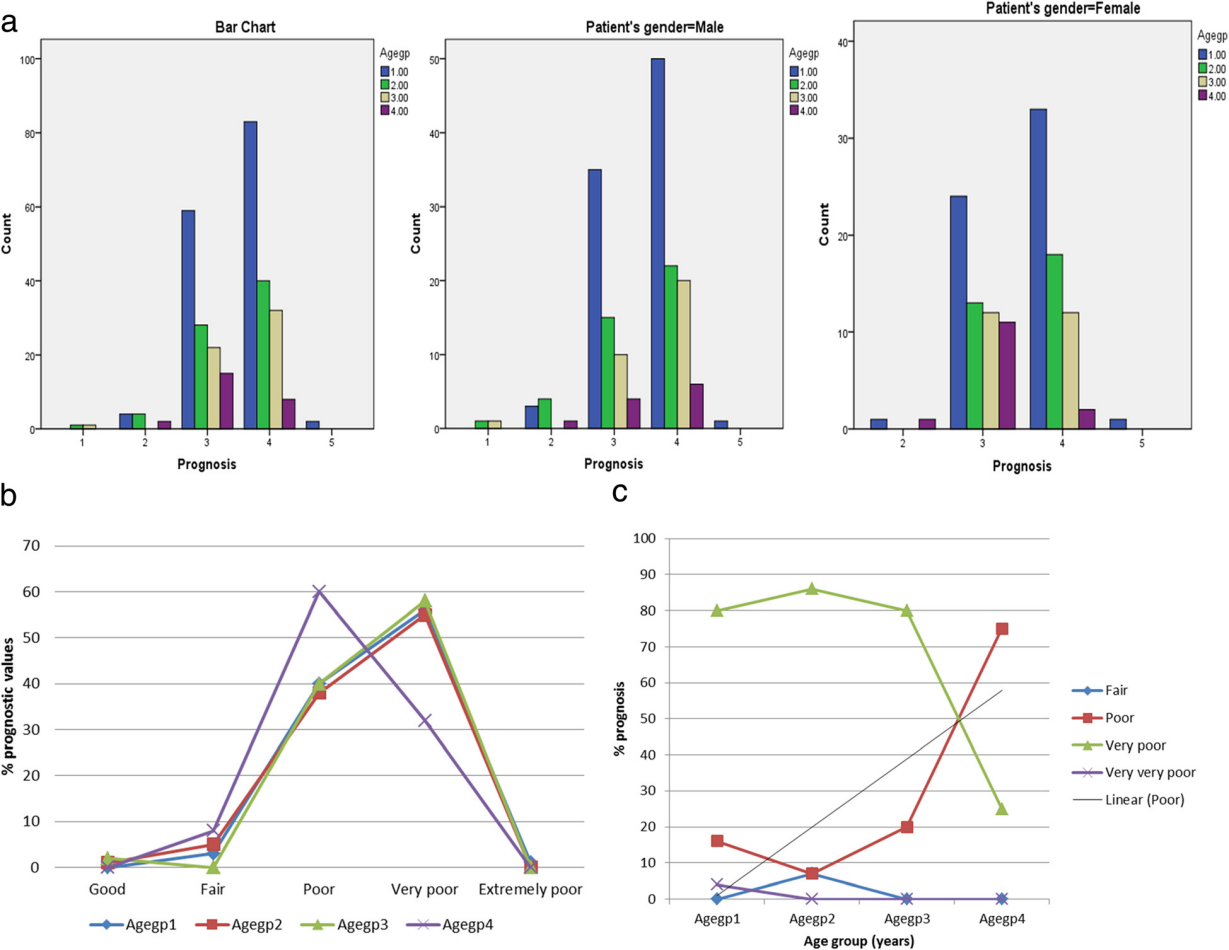
**(a). Prognosis of under-fives in ICU (i) all patients relative to age group (ii).** Prognosis of under-fives in ICU (ii) male patients relative to age group. iii. Prognosis of under-fives in ICU female patients relative to age group (**b**). Per cent prognostic values relative to age group of children admitted into ICU. (**c**) Percent prognostic values among children that died in the ICU.

Prognostic values ranged mainly from poor to very poor across all age groups. Table 6 and Figure 4a (i) show that only 2 (0.7%) of the 301 entrants into ICU had good prognosis among whom none died. These two children were males (Figure 4a (ii)). Among females, there was no significant difference in prognostic values within the age range of 2.1 and 4.9 (Figure 4a (iii)). In all, 124 (41%) and 163 (54%) had poor and very poor prognostic values respectively (Figure 4(b) among whom 11 (9%) and 46 (28%) died (Table 6). Among those that died, prognostic values were poor and very poor for 16% and 81% of children aged 1-1.9 years, for 7% and 86% of those aged 2-2.9 years and for 20% and 80% among those aged 3-3.9 years. This trend was reversed for children aged 4-4.9 years into 75% (poor prognosis) and 25% (very poor prognosis) respectively (Figure 4(c)).

**Figure 5:**
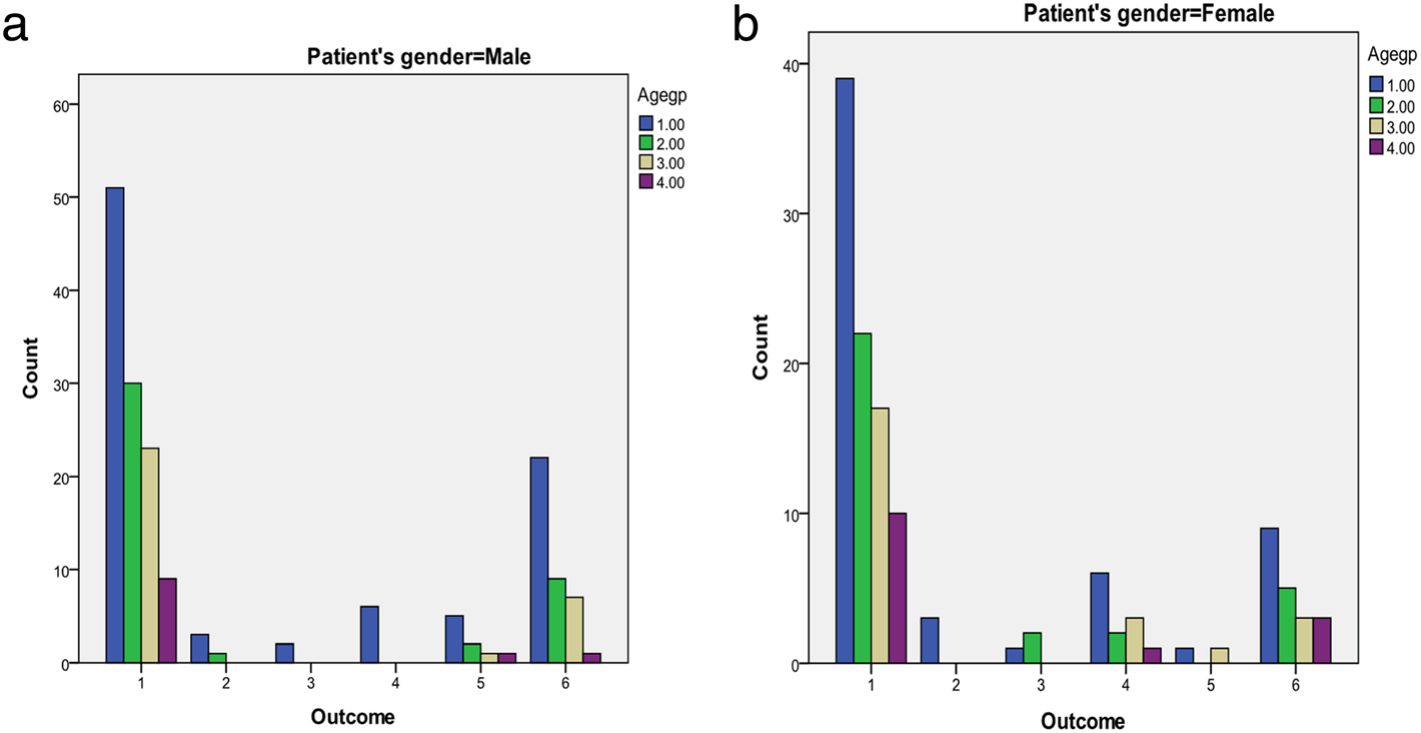
a. Outcome of male admission into intensive care unit according to age group. b. Outcome of female admission into intensive care unit according to age group.

### Outcome of admission into ICU

The outcome of admissions into the ICU for all the children in different age groups and gender is as indicated in Table 7 and Figure 5. Of the 301 children admitted into the ICU, 201 (66%) recovered fully and were discharged home but 59 (19.0%) died. Interestingly almost 6% of the children (4% males, 9% females) were discharged against medical advice and 7 (2.3%) absconded. Overall, 76% of deaths (80% in males, 70% in females) occurred between the age groups of 1-1.9 and 2- 2.9 years. In general, children aged 1-1.9 years were about 1Â½ times more likely to die in ICU than to recover fully and be discharged home (OR = 1.37 [0.73, 2.54]) and of these, males were over 1Â½ times more likely to die in ICU than to be discharged (OR 1.57 [0.7-3.5]).

**Table 7:** Outcome of children admission into the ICU

	**Age group (years)**			
	1-1.9	2-2.9	3-3.9	4-4.9
***Outcome***	(n=148)	(n=73)	(n=55)	(n=25)
***All childrenadmitted intoICU***				
- Fully recovered and discharged*	90 (61)	52 (71)	40 (73)	19 (76)
- Absconded	6 (4)	1 (1)	0 (0)	0 (0)
- Referred	3 (2)	2 (3)	0 (0)	0 (0)
- DAMA	12 (8)	2 (3)	3 (5)	1 (4)
- On admission	6 (4)	2 (3)	2 (4)	1 (4)
- Died*	31 (21)	14 (19)	10 (18)	4 (16)
Odds ratio	1.37	0.89	0.82	0.70
CI	0.7-2.5	0.4-1.8	0.4-1.9	0.2-1.9
***Male childrenadmitted intoICU***				
	(n=89)	(n=42)	(n=31)	(n=11)
- Fully recovered and discharged*	51 (57)	30 (71)	23 (74)	9 (82)
- Absconded	3 (3)	1 (2)	0 (0)	0 (0)
- Referred	2 (2)	0 (0)	0 (0)	0 (0)
- DAMA	6 (7)	0 (3)	0 (0)	0 (0)
- On admission	5 (6)	2 (5)	1 (3)	1 (9)
- Died*	22 (25)	9 (21)	7 (19)	1 (9)
Odds ratio	1.57	0.83	0.86	0.30
CI	0.7-3.5	0.3-2.1	0.3-2.4	0.01-2.5
***Female childrenadmitted intoICU***				
	(n=59)	(n=31)	(n=24)	(n=14)
- Fully recovered and discharged	39 (66)	22 (71)	17 (71)	10 (71)
- Absconded	3 (5)	0 (0)	0 (0)	0 (0)
- Referred	1 (2)	2 (6)	0 (0)	0 (0)
- DAMA	6 (10)	2 (6)	3 (13)	1 (7)
- On admission	1 (2)	0 (0)	1 (4)	0 (0)
- Died	9 (15)	5 (16)	3 (13)	3 (22)
Odds ratio	1.0	1.0	0.74	1.4
CI	0.4-3.0	0.3-3.4	0.2-3.1	0.3-6.3

*Odds ratio was calculated for “fully recovered and discharged” and for “died”. ( )=%; CI=Confidence Interval.

DAMA=Discharged against medical advice.

Table 8 indicates correlations between variable of the children in the ICU. Patient’s age correlated positively with the primary diagnosis (r = 0.116 p = 0.45), and patient’s weight correlated positively with first diagnosis (r = 0.132, p = .022) and negatively with prognosis (r = -0.143, p = 0.013) and negatively with outcome (r = -0.195, p = 0.001).

**Table 8:** Correlation coefficients of some variables of the study children

	**Patient's age**	**Patient's gender**	**Patient's weight (kg)**	**Patient's temperature**	**1st diagnosis**			**Age group**
Patient’s age	1	.064	.727	-.027	.116*	-.109	-.058	.969**
		.272	.000	.636	.045	.058	.318	.000
	301	301	301	301	301	301	301	301
Patient’s gender	.064	1	-.041	.042	-.020	-.007	-.078	.076
Sig. (2-tailed)	.272		.484	.463	.727	.902	.179	.186
N	301	301	301	301	301	301	301	301
Patient’s weight (kg)	.727	-.041	1	-.025	.132*	-.143	-.195	.721**
	.000	.484		.669	.022	0.13	.001	.000
	301	301	301	301	301	301	301	301
Patient’s temperature	-.027	.042	-.025	1	-.070	-.098	-.042	-.016
	.636	.463	.669		.223	.090	.463	.779
	301	301	301	301	301	301	301	301
1st diagnosis	.116	-.020	.132	-.070	1	-.066	.028	.121*
Sig. (2-tailed)	.045	.727	.022	.223		.257	.631	.036
N	301	301	301	301	301	301	301	301
Prognosis	-.109	-.007	-.143	-.098	-.066	1	.196	-.102
	.058	.902	.013	.090	.257		.001	.079
	301	301	301	301	301	301	301	301
Outcome	-.058	-.078	-.195	-.042	.028	.196	1	-.075
Sig. (2-tailed)	.318	.179	.001	.463	.631	.001		.194
N	301	301	301	301	301	301	301	301
Age group	.969	.076	.721	-.016	.121*	-.102	-.075	1
	.000	.186	.000	.779	.036	.079	.194	
N	301	301	301	301	301	301	301	301

**. Correlation is significant at the 0.01 level (2-tailed).

*. Correlation is significant at the 0.05 level (2-tailed).

## Discussion

The poor health outcomes in Nigeria mainly stem from a lack of appropriate targeting strategies for reaching poor and under-served populations and low levels of public funding [18]. Historical case studies [19] and contemporary analysis of various political systems [20,21] have shown that governments are most successful in promoting health when they invest in social protection and create health-promoting environments [5]. There have been ample evidences to indicate that the capacity of the health care sector to improve population health and health equity is strongly influenced by other sectors [22,23]. A major, but often unseen, apprehension on why high child mortality exists in Nigeria, as shown by our data, could be explained by the inverse care law [24] in which, though it is recognized that access to health care is a crucial social determinant of health, those with the worst health conditions receive the least health care. The poor and excluded groups are in poorer health conditions than are their richer counterparts but are less able to access or benefit from care [5]. A very important finding in this study is that children with reduced weight are about three and a half times more likely to die in ICU than to recover and be discharged home. Studies have shown that children bear the brunt of poor socio-economic planning evidenced [3] and those under the age of five years had more than four times the mortality burden of the rest of the population [4]. This agrees with data from this study which further stresses that low body weight, a result of poor nutrition, apparently plays a central role in child mortality in SSA. This supports the assertion that under-nutrition is associated with an increased risk of death among young children in developing countries [25]. That 90% of under-five mortality occurred within the weight range of 5.1 and 15.0 kg is a clear pointer to the fact that malnutrition is fundamentally contributory to the death of these children. Availability of nutritious food, good infant feeding habits and mother's education are strong reasons to bring together line ministries such as Health, Agriculture, Education, Women Affairs, Youth and Social Services and Finance. The notion that there should be specific places for children to play and toys to play with are literarily non-existent. Although 67% of those that were admitted into the ICU got better and were discharged home, these children would return to the same poor environmental and poor family conditions from where they got ill initially; their parents would have parted with a large sum of hard-earned out-of-pocket expenses to pay for their wards admission and thereby becoming poorer because of lack of a viable health insurance. A considerable body of evidence exists describing the vital importance of social and economic factors at a collective societal level that directly determine population health and health equity [5]. Studies have also provided evidence that life expectancy is linked more to improved living conditions than to improved health care services [19,26,27]. The death of 59 out of 301 children recorded in this study translates to an unacceptably high child mortality rate of 196/1000. To address this issue of high child mortality, a call is hereby made for health reform in Nigeria to pay increased attention to social and behavioural determinants of health over unilateral pursuit of health care reform such as building big hospitals. Among nations of the OECD, increased social spending is linked to lower infant (*and child*) mortality and longer life expectancy [20]. It is observed that between 2003 and 2005, the Scandinavian countries of Sweden, Denmark and Norway spent only 9.1% respectively of their GDP on health care but 31.3%, 27.6% and 25.1% respectively of their GDP on social investments and came up with infant mortality rates of 2.4, 4.4 and 3.1 per 1000 live births respectively [28]. The better outcome in health indices of Europeans are more likely a result of greater emphasis on social policy [28]. Nigeria has achieved a lot of progress in various sectors of governance, but it still faces significant challenges in accelerating growth, reducing poverty and meeting the Millennium Development Goals (MDG) [18], an observation made elsewhere [10]. For example, in 2003, only 3.3% of Nigeria’s total budget was allocated to health. General public services and economic affairs together made up more than 50% of Nigeria’s consolidated government expenditure in 2010, with spending on social sectors averaging 20% over the period 2005-2010. Of the social sectors, education made up approximately 12% of total government expenditure in 2009, health around 7% and social protection only about 1.4% [19]. The World Health Organization reiterates that most of the health and developmental challenges in Nigeria have not changed significantly and that Nigeria is on track toward achieving, in part or in whole, only three out of the eight MDGs, namely, basic education, HIV prevalence and the global partnership for development [29] excluding MDG4. Interventions such as Reaching Every Ward (REW), Integrated Management of Childhood Illness (IMCI) Strategy, and Integrated Maternal Neonatal and Child Health (IMNCH) strategy cannot lead to the achievement of MDG 4 without basing them on the social determinants of health.

The National Health Insurance Scheme (NHIS), officially launched in the country in June of 2005, is worth mentioning here. NHIS was expected to boost funds flowing into the healthcare sector in which workers pay up to 5% of their salary into the scheme in order to qualify for free treatment for themselves, a spouse and up to four children. In the end, poor planning, lack of research to provide evidence-based infrastructure as well as lack of accountability spelt the doom of NHIS, as there was no particularly viable health scheme for the teeming population of Nigerians not working for the government. Despite being relatively rich compared with other countries in sub-Saharan Africa in terms of per capita GDP, Nigeria spends a significantly lower share on social protection, less than 1% in 2006-2007; Ethiopia, Kenya, Malawi, Mozambique and Uganda spent an average of 1.4% in the same year [9].

What could have been done to save the children? The answer lies beyond health reforms and extends into government policies on housing, roads, provision of basic amenities, adequate transportation, mechanized agriculture and elimination of health inequity. Factors such as growing income inequality, economic and social dislocations caused by internal conflicts, relative shortage of social and economic infrastructure in rural areas, low productivity especially agriculture, migration of the educated work force from the rural to the urban areas and the brain-drain syndrome may directly or indirectly contribute to the observed high child mortality. Government hospitals should emulate their North or South African counterparts to adequately provide for those children (and adults) who would require intensive care; but more especially, government should focus more attention on its social policies. This emphasizes that reform in Nigeria’s health care delivery, access and funding are certainly needed. Meaningful improvement in population health may require the country to invest as heavily in social programs as the Scandinavian countries. Social policies that care for people are the best investments for health [28] and investing in social determinants of health will be a wise and cost-effective economic spending that can save the country a lot in terms of funds and human lives. Concentrating only on health care reform to reduce child mortality is not only counter-productive but also encourages lack of transparency, moves Nigeria farther away from achieving MDG4 by 2015, cannot reduce infant, child and maternal mortality as rapidly as desired but will probably reduce life expectancy further due to continued galloping child mortality rate.

### Limitations

This study has its own limitations. First, data that were analyzed were collected retrospectively, therefore the accuracy of these data cannot be fully ascertained, though there is a high index of credibility that they were recorded according to the events that took place at the ICU when the children were admitted. Secondly, the instrument for collecting the data were not standardized according to Cronberg’s alpha. Thirdly, causes of deaths were not categorized according to the International Classification of Diseases, 10th revision (ICD-10; webappendix pp 1—3). Fourth, criteria for prognostic differentiation of the children were not defined. Fifth, the result from the study may not be generalized to the entire population of Nigeria. Sixth, exact causes of death of the study children were not determined and finally, though the study was not particularly on social determinants of health, inferences were made to them.

## Conclusion

Child mortality is still high in Nigeria. The government should set up a body of researchers to critically appraise social determinants of health and health inequity. A lot still has to be done to ameliorate the incessant onslaught of the negative outcomes associated with social determinants of health and health inequity for the country to join the league of healthy nations.

### Recommendations

The Federal Government of Nigeria is doing all things possible to improve the health of its people. In furtherance to this, it should establish the Healthy People 2015 Initiative as a goal towards the elimination of health disparities among Nigerians. In addition, the government should make a large investment in research into the health disparities and health equity, targeting multi-agency collaboration which should include representatives of Civil Society Organization and Labour Congress. There is a need for more innovative approaches derived from trans-disciplinary research and involving multiple Federal Ministries and different disciplines such as Health, Finance, Women Affairs, Water Resources, Communication, Transportation, Environment, Youth and Social Services and others. Also, the Federal Ministry of Health, as a matter of urgency, should establish Health Promotion Division to be headed by a professional in this area. State Ministries of Health should also emulate this planned suggestion.

## Competing interests

The authors declare that they have no competing interests.

## Authors’ contributions

BMA and CC conceptualized the study and organized the analytic plan. AE supervised all aspects of data collection and implementation of the study. BMA, AE and DD conducted the analyses. BMA led the writing and all authors contributed to the interpretation of the findings and the writing. All authors read and approved the final manuscript.

## About the authors

Bamgboye M. Afolabi is the Chief Executive Director of Health, Environment and Development Foundation, a Non-Governmental Organization with its office in Lagos Nigeria, Cecilia Clement is with the Department of Morbid Anatomy, Lagos University Teaching Hospital, Idi-araba, Lagos; Adejuwonlo Ekundayo is the Director of Silverwood Consult, a Research and Development outfit in Lagos, Nigeria and Duro Dolapo is with the Support for National Malaria Program (SuNMaP), Abuja, Nigeria.
